# Improvising through a pandemic: adapting a medical improv elective over Zoom

**DOI:** 10.5116/ijme.6183.b4ef

**Published:** 2021-11-26

**Authors:** Nicholas Neel, John-Michael Maury, Lina Lander

**Affiliations:** 1 University of California, San Diego School of Medicine, San Diego, CA, USA; 2 Professional Development Center, Division of Medical Education, School of Medicine, University of California San Diego, CA, USA; 3 Department of Family Medicine, University of California, San Diego, CA, USA

**Keywords:** Medical improv, medical education, remote learning, curriculum development

## To the Editor

Born out of improvisational theatre, medical improv takes the basic ideas of improv (listening, acceptance, adaptability, teamwork, and resiliency) and applies them to clinical situations.^1^ Throughout the country, there have been a handful of medical schools that have implemented some form of improv training into their curriculum.^2^^,^^3^ These courses have shown great promise in enhancing students' communication skills as well as providing an enjoyable environment for learners.^4^^, ^^5^

Our institution launched its inaugural medical improv elective in 2019, inspired by Katie Watson's work.^6^  Our elective was crafted after training with Watson and adopting many aspects of her curriculum for medical students. The course mainly focused on experiential learning, where students participated in improv exercises followed by debriefing sessions where lessons learned in improv were applied to some aspect of clinical training. Improv, however, relies heavily on in-person activities, emphasizing both verbal and non-verbal communication. Exercises require interplay among participants reacting to others' responses, emotions, and body language, making physical interaction a major aspect of the learning and development taking place in improv.

The COVID-19 pandemic dramatically impacted medical students across the country. One unique challenge our institution faced was the adaptation of a medical improv course to be fully remote. Given the success of previous in-person medical improv classes and the immense benefit felt by students participating in the course, we decided to "Yes, and" this unique experience and incorporate the spirit of improv into a remote synchronous elective.

In order for improv to successfully function, it requires a basic set of rules for performers to follow. Despite many different interpretations of "The Rules of Improv", the fundamental principles are consistent. We chose the following, simplified, three rules to guide our class: 1. Attune (listen), 2. Affirm then Add ("Yes, and…"), and 3. Adhere (commit).

 These guiding principles direct students back to the core of improv, centering on listening to themselves and those around them, actively contributing, and committing headfirst without fear or trepidation into the scene or situation at hand. During the course, students regularly reflected on how these tenets might pertain to them, both personally and professionally.

Adapting a physical, experiential activity, such as improv, into a 2-dimensional platform presented unique challenges. We used the video conferencing software, Zoom, provided by our institution. We applied the same tenets we teach to our present situation. We attuned to the remote environment of Zoom and utilized available functionality. Because many of the exercises we normally share in class would not work virtually, we utilized a few simple methods to shift and meet our learning objectives.

Exercises and scene work was modified to provide optimal interaction over videoconferencing software. For example, Zip, Zap, Zop, a favorite warm-up exercise, required revamping to function virtually. In person, learners point to each other and pass the "energy", saying the correct word of the pattern in a specific order, Zip, Zap, then Zop. This sequence is random as learners pass the pattern anywhere in the circle requiring physical connection through a pointed finger and focused, direct eye contact. The pattern continues until a player says the wrong word, hesitates or speaks a word that does not exist in the pattern. We modified the pattern to two words to create a quicker 'give and take' over Zoom. Instead of Zip, Zap and Zop, we used "Zing" and "Pop" and directed players to pantomime passing an imaginary ball when they tossed the energy to another player. In the remote version, students quickly named the player to whom they were throwing the energy and pantomimed the pass while saying "Zing". The player named would then acknowledge the reception of the gift by physically acting out, catching the ball and saying "Pop". This modified version gave the excitement of the speed of an in-person version of Zip, Zap, Zop without the extra hurdles and hiccups. Players have to remain aware of the virtual visual field and welcome the energy at any given moment.

Like most improvisational workshops, we incrementally worked toward building strong, solid scenes over the course of our elective. Given the lack of shared physical space due to Zoom, we had to improvise techniques to simulate students participating in a scene together. The Video ON/OFF feature of Zoom became a tool for students to simulate walking in and out of a scene. In addition, we encouraged students to pantomime into the corners of their screen as a way to mimic something being given or tossed to another player. Lastly, seeing oneself on the screen allowed students to see their facial expressions as they portrayed emotions giving them real-time feedback as they could see how they were received by others in the moment.

Eleven first- and second-year medical students participated in the course and provided feedback on their experience through course evaluations. Student responses were overwhelmingly positive, with several students commenting that the class helped them through the COVID-19 pandemic. The themes addressed included the formation of relationships with students despite distance learning, providing a break from the stress of medical school, and improving communication in patient interactions. Many also commented on how the lessons and skills they gained from improv would be beneficial to them as future physicians.

While many courses had to adapt to an online format due to the COVID-19 pandemic, humanities-focused electives were slower to follow suit.^7^ We describe one example of a medical improv elective being facilitated over Zoom. With a few modifications, other universities can incorporate improv into their distance-learning curriculum. The benefits of improv are immense, and its application in medical education has shown promise in improving student communication, empathy, and teamwork.^4^^,^^5^ We have seen this firsthand at our institution, with many students thoroughly enjoying their experience and confirming personal benefit gained from participation. The challenges faced due to COVID-19 provided a unique environment to practice the principles of improv and attune, adapt, and commit to the situation at hand. Students who took the improv elective over Zoom described how improv enhanced their communication and adaptability skills, and some also touched upon the benefits it had in their social lives and personal wellbeing. We must state that online improv via Zoom does not replace an in-person experience but is an acceptable alternative. Improv was a wonderful tool for virtually connecting with other students during a global pandemic. This course became a highlight of the week for facilitators and many students. Now, more than ever, this art form was needed to help participants feel a sense of community and connection. It is our hope that this further bolsters the importance and relevance of improv in the training of medical students across the country.

### Conflicts of Interest

The authors declare that they have no conflict of interest.  

## References

[1] Watson K, Fu B (2016). Medical improv: a Novel approach to teaching communication and professionalism skills.. Ann Intern Med.

[2] Shochet R, King J, Levine R, Clever S, Wright S (2013). 'Thinking on my feet': an improvisation course to enhance students' confidence and responsiveness in the medical interview.. Educ Prim Care.

[3] Hoffman A, Utley B, Ciccarone D (2008). Improving medical student communication skills through improvisational theatre.. Med Educ.

[4] Cai F, Ruhotina M, Bowler M, Howard E, Has P, Frishman GN, Wohlrab K (2019). Can I get a suggestion? Medical improv as a tool for empathy training in obstetrics and gynecology residents.. J Grad Med Educ.

[5] Krusen NE (2012). Improvisation as an adaptive strategy for occupational therapy practice.. Occup Ther Health Care.

[6] Watson K (2011). Perspective: Serious play: teaching medical skills with improvisational theater techniques.. Acad Med.

[7] Shahrvini B, Baxter SL, Coffey CS, MacDonald BV, Lander L (2021). Pre-clinical remote undergraduate medical education during the COVID-19 pandemic: a survey study.. BMC Med Educ.

